# Patients satisfaction in an academic walk-in centre: a new model of residents training achieved by family doctors

**DOI:** 10.1186/1756-0500-7-874

**Published:** 2014-12-04

**Authors:** Ismail Labgaa, Isabella Locatelli, Thomas Bischoff, Willy Gilgien, Philippe Staeger, Jacques Cornuz, Jean Perdrix

**Affiliations:** Policlinique Médicale Universitaire, Rue du Bugnon 44, CH-1011 Lausanne, Switzerland; Permanence PMU-FLON, Bâtiment Les Mercier A, Voie du Chariot 4, CH-1003 Lausanne, Switzerland

**Keywords:** Patients satisfaction, Medical education, Family Medicine, Walk-in, Residency A part of the results was presented at the 11th Congress of CNGE, Bordeaux, France, November 2011

## Abstract

**Background:**

Walk-in centres may improve access to healthcare for some patients, due to their convenient location and extensive opening hours, with no need for an appointment. Herein, we describe and assess a new model of walk-in centre, characterised by care provided by residents and supervision achieved by experienced family doctors. The main aim of the study was to assess patients’ satisfaction about the care they received from residents and their supervision by family doctors. The secondary aim was to describe walk-in patients’ demographic characteristics and to identify potential associations with satisfaction.

**Methods:**

The study was conducted in the walk-in centre of Lausanne. Patients who consulted between 11th and 31st April were automatically included and received a questionnaire in French. We used a five-point Likert scale, ranging from “not at all satisfied” to “very satisfied”, converted from values of 1 to 5. We focused on the satisfaction regarding residents’ care and supervision by a family doctor. The former was divided in three categories: “Skills”, “Treatment” and “Behaviour”. A mean satisfaction score was calculated for each category and a multivariable logistic model was applied in order to identify associations with patients’ demographics.

**Results:**

The overall response rate was 47% [184/395]. Walk-in patients were more likely to be women (62%), young (median age 31), with a high education level (40% of University degree or equivalent). Patients were “very satisfied” with residents’ care, with a median satisfaction score between 4.5 and 5, for each category. Over 90% of patients were “satisfied” or “very satisfied” that a family doctor was involved in the consultation. Age showed the greatest association with satisfaction.

**Conclusion:**

Patients were highly satisfied with care provided by residents and with the involvement of a family doctor in the consultation. Older age showed the greatest positive association with satisfaction with a positive impact. The high level satisfaction reported by walk-in patients supports this new model of walk-in centre.

**Electronic supplementary material:**

The online version of this article (doi:10.1186/1756-0500-7-874) contains supplementary material, which is available to authorized users.

## Background

A walk-in centre is characterised by its convenient location with extensive opening hours and the opportunity to be cared for by a health professional without an appointment. Therefore walk-in centres may improve the accessibility to a healthcare system [1, 2]. Walk-in centres have been implemented in North America and the United Kingdom (UK) for decades and only recently in Switzerland.

We conducted a survey in a walk-in centre led by the University Hospital. This walk-in centre represents a new model characterised by care provided by young residents. While in most walk-in centres, patient care is provided by nurses or physicians; in this walk-in centre, patient care is provided by residents and experienced family doctors. Any patient health issue is first managed by a resident; then, an experienced family doctor is involved by supervising and teaching the resident. Although academic outpatient clinics have been described in Switzerland and in other countries [3, 4], this system is described for the first time herein, in a walk-in setting. The main aim of the study was to assess patients’ satisfaction with care provided by residents and with the involvement of a family doctor.

Patients’ satisfaction has been used as an indicator of performance of healthcare systems [5, 6] but remains a questionable indicator for quality of received care [7, 8]. Moreover, patients’ satisfaction may have an impact on major outcomes. Previous studies have demonstrated the impact of satisfaction on adherence to treatment, which may contribute to better care [9, 10].

Global satisfaction is mainly associated with receiving the expected medical care and being treated well by a doctor [11].

We also aimed to describe patients’ demographics and to identify if there are associations between demographics and satisfaction.

## Methods

The study was conducted at the *Permanence PMU-FLON* of Lausanne, a walk-in centre managed by the *Policlinique Médicale Universitaire*. The data collection was performed between April 11th and April 31st 2011. Patients consulting during the study period were automatically included and received postal correspondence explaining the study purpose with the questionnaire and a pre-paid return envelope. Non-respondents were sent an identical study pack, two weeks after the initial mailing.

The questionnaire assessed patients’ characteristics and their satisfaction regarding practical aspects of the walk-in centre (data not shown) and regarding received care. Questions were taken or adapted from existing validated questionnaires [12–14]. A five-point Likert scale was used for questions about satisfaction. The scale, from “not at all satisfied” to “very satisfied”, was numerically converted from 1 to 5 [15].

We performed descriptive statistical analyses for the socio-demographic characteristics.

Analyses were focused on patient satisfaction about received care, which included satisfaction with care provided by residents and satisfaction with supervision by a family doctor. The former was assessed using 13 items while the latter was assessed by a single item, investigating whether patients were satisfied with the involvement of a family doctor in the consultation. Care provided by residents was divided in three categories: “Skills”, “Treatment” and “Behaviour”. These categories and their respective items are detailed in Additional file 1: Table S1.

Satisfaction about the three above mentioned categories was calculated as a mean satisfaction score about the concerned items. For instance, a subject reporting to be “Satisfied” (=4) for items “Attention”, “Clinical assessment” and “Communication”; and “Very satisfied” (=5) for “Explanations”, had a global (mean) satisfaction for the Skills category of (4 + 4 + 4 + 5)/4 = 4.25. In case of missing item in a category, the satisfaction was calculated as the mean of non-missing items in this category. For each satisfaction category, we also constructed a dichotomous variable, taking value one whether a patient reported “very satisfied” to all items of the category, and zero otherwise. A multivariable logistic model was applied, in order to determine associations of the above dichotomous variable with socio-demographic characteristics for each satisfaction category. In the model, a weight was attributed to each patient proportionally to the number of non-missing items defining the satisfaction. Moreover, a sensitivity analysis was performed in order to test the robustness of our results with respect to a different dichotomization of the satisfaction variable. In this analysis we defined satisfaction as taking value one if the mean satisfaction over the different items defining the category was greater than 4, i.e. the patient was at least “satisfied” on average of the respective items. We used R program (v 2.11.1, http://www.r-project.org/) for descriptive statistics, graphics, and logistic models.

All study related data were anonymously treated. The study received the approval from the ethics committee of the State of Vaud, Switzerland (Protocol 492/13).

## Results

### Socio-demographic characteristics

The overall patients’ response rate was 47% (184/395). Walk-in patients were characterised by a preponderance of women (62%), Swiss citizens (65%), and young patients (median age 31). Respondents were more likely to be women with respect to non-respondents (71% vs. 54%), to have Swiss citizenships (72% vs. 58%) and to be older (median age 36 vs. 28). Married patients accounted for 29%, without statistical difference between respondents and non-respondents. Information about education degree and working condition was only available for respondent: 40% of respondent patients had a high education level (university or equivalent) and more than 80% were working or student (Additional file 2: Table S2).

### Patients’ satisfaction with care provided by residents

**Figure 1:**
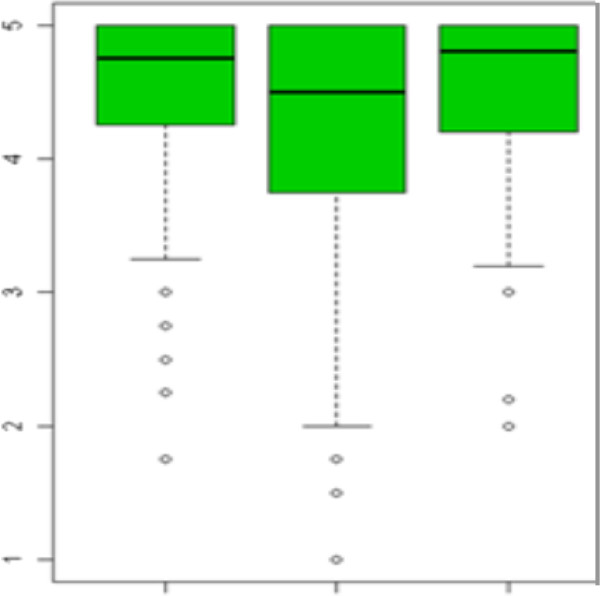
**Patient satisfaction with care provided by residents, divided in three categories: “skills”, “treatment” and “behaviour”.**

As described in Additional file 3: Table S3, satisfaction with care provided by residents was divided in three categories: “skills”, “treatment” and “behaviour”. Figure 1 shows the satisfaction distribution for each category. All distributions were strongly asymmetric to the left, with medians between 4.5 and 5, that corresponds to a satisfaction level between “satisfied” and “very “satisfied”.

### Patients’ satisfaction with supervision of residents by family doctors

**Figure 2:**
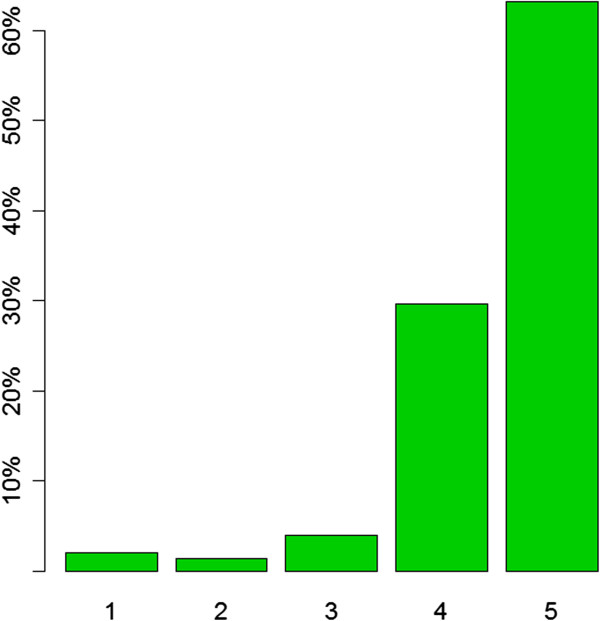
**Patients’ satisfaction with supervision of residents by experienced family doctors (1: not at all satisfied, 2: not satisfied, 3: partially satisfied, 4: satisfied, 5: very satisfied).**

More than 95% (141/152) of walk-in patients were “satisfied” or “very satisfied” that a family doctor had taken part in the management of their health issue (Figure 2).

### Associations between patients’ demographics and satisfaction

Studied characteristics were: age, gender, education, profession and nationality. Older age, female gender and higher education were the patients characteristics associated with higher satisfaction (Additional file 3: Table S3). Herein, satisfaction was re-defined as a dichotomous variable, taking value one if a patient was “very satisfied” on *each* item defining the category, and zero otherwise. For gender, we noted a trend for women being more frequently “very satisfied” than men (OR >1), with a statistically significance for the supervision system (OR: 2.7; 95% CI: 1.25-5.97). Older patients reported higher satisfaction levels than younger ones (ORs between 1.35 (Social skills) and 1.48 (Treatment), with 10 years age unit). We also highlighted an association between education level and higher satisfaction. Patients who received a high level of education showed a trend towards higher satisfaction. This positive association was significant for “skills” (OR: 2.06; 95% CI: 1.06-4.16) and “treatment” (OR: 2.77; 95% CI: 1.30-6.29), while a trend was observed for “supervision system” (OR: 2.09; 95% CI: 0.99-4.53). Although dissatisfaction’s rate was law (16% on average), the results of our sensitivity analysis were comparable to the ones shown in Additional file 3: Table S3.

## Discussion and conclusions

This study described a unique and innovative system of walk-in centre with care provided by residents taught by experienced family doctors. Patients reported high levels of satisfaction with care provided by residents. They were also satisfied with the involvement of a family doctor in the consultation. Age, gender and level of education showed associations with high satisfaction, which was described in other studies [16].

Our study has some limitations. Although generally comparable to respondents, non-respondents displayed a lower rate of women, a lower rate of Swiss citizenship and a younger median age. Hence, sampling bias may have been introduced. Furthermore, patients who did not speak French could not respond to the questionnaire, which could have also led to selection bias. The rate of missing data was acceptable. Moreover, missing data were considered in the multivariable model by attributing a weight based on the number of non-missing data composing the category.

In a review of the literature about walk-in centres in 2003, *Salisbury C and Munro J* described similar demographic characteristics, with walk-in patients more likely to be women, young and in some form of employment [1]. Studies reported high levels of satisfaction among walk-in patients in the United States (USA) [17, 18], Canada [19–21] and in the UK [22]. Patients’ satisfaction reported in our model is comparable to the ones described in these studies. Therefore we can assume that our model of walk-in centre with care provided by residents is an interesting alternative to nurse or physicians led walk-in centres. Patients’ satisfaction appears to be strongly associated with care provided by medical staff and their behaviour [11, 23].

Other studies showed similar associations with age [16] gender [24–26] and education [27, 28].

### Potential applications in clinical practice

Walk-in centres may improve the access to healthcare for some patients [1, 2]. They appeared to be particularly attractive to women, young people and patients in employment. Reported satisfaction was high which supporting the relevance of this type of walk-in centre, with care provided by residents and supervision by experienced family doctors.

In conclusion, patients were highly satisfied with care provided by residents and with the involvement of a family doctor in the consultation, that are the characteristics of this new model of academic walk-in centre. Older age was the major association on satisfaction with a positive impact.

## Electronic supplementary material

Additional file 1: Table S1: Categories of items assessing patients satisfaction for care provided by residents. (PDF 372 KB)

Additional file 2: Table S2: Socio-demographic characteristics of walk-in patients. (PDF 291 KB)

Additional file 3: Table S3: Impact of gender, age and education level on satisfaction as a dichotomous variable (1=“very satisfied” on each item defining the category). (PDF 290 KB)
