# Supplementary material for: Patients satisfaction in an academic walk-in centre: a new model of residents training achieved by family doctors
Source: BMC Res Notes. 2014 Dec 4;7:874. doi: 10.1186/1756-0500-7-874 (PMC4295283; doi:10.1186/1756-0500-7-874)
Supplement: Supplementary file 1 — Additional file 1: Table S1: Categories of items assessing patients satisfaction for care provided by residents. (PDF 372 KB) [file 13104_2014_3467_MOESM1_ESM.pdf]

Category	Concerns		Nb of items
Skills	Attention<br>Clinical assessment<br>Communication<br>Explanations		4
Treatment	Reasons for<br>How to take<br>Side effects of<br>Alert signs of<br>Involvement in<br>decision-making for	the treatment	5
Behaviour	Kindness<br>Confidence<br>Respect<br>Time spent		4

Table 1: categories of items assessing patients' satisfaction for care provided by residents
