# Supplementary material for: Patients satisfaction in an academic walk-in centre: a new model of residents training achieved by family doctors
Source: BMC Res Notes. 2014 Dec 4;7:874. doi: 10.1186/1756-0500-7-874 (PMC4295283; doi:10.1186/1756-0500-7-874)
Supplement: Supplementary file 2 — Additional file 2: Table S2: Socio-demographic characteristics of walk-in patients. (PDF 291 KB) [file 13104_2014_3467_MOESM2_ESM.pdf]

	Respondents (n = 184)	Non-respondents (n = 211)	p of the difference	Overall (395)
**Age y, median (IQ range)**	36 (26-51)	28 (24-36)	< 0.001*	31 (25-43)
**Under the age of 50 years, n (%)**	135 (73.4)	198 (93.8)	< 0.001$	333 (84.3)
**Men, n (%)**	54 (29.4)	96 (45.5)	< 0.001$	150 (38.0)
**Swiss citizenships, n (%)**	131 (72.0)	122 (58.4)	0.005$	253 (64.7)
**Married, n (%)**	59 (32.2)	53 (25.2)	0.124$	112 (28.5)
**High education University degree or equivalent, n (%)**	71 (40.1)			
**Working or at training (ic student), n (%)**	145 (81.0)			

*Mann-Withney-Wicoxon test ; $Chi square test

Table 2: socio-demographic characteristics of walk-in patients
