# Supplementary material for: Patients satisfaction in an academic walk-in centre: a new model of residents training achieved by family doctors
Source: BMC Res Notes. 2014 Dec 4;7:874. doi: 10.1186/1756-0500-7-874 (PMC4295283; doi:10.1186/1756-0500-7-874)
Supplement: Supplementary file 3 — Additional file 3: Table S3: Impact of gender, age and education level on satisfaction as a dichotomous variable (1=“very satisfied” on each item defining the category). (PDF 290 KB) [file 13104_2014_3467_MOESM3_ESM.pdf]

**Satisfaction categories**		**Gender (women)**			**Age (+10 years)**			**Education (High)**		
	**satisfied**	**OR**	**95% CI**	**pvalue**	**OR**	**95% CI**	**pvalue**	**OR**	**95% CI**	**pvalue**
**Skills (n=183)**	47%	1.30	(0.65; 2.63)	0.465	1.45	(1.18; 1.82)	0.001	2.06	(1.06; 4.16)	0.037
**Treatment (n=167)**	34%	1.54	(0.72; 3.45)	0.280	1.48	(1.18; 1.91)	0.001	2.77	(1.30; 6.29)	0.011
**Behaviour (n=184)**	40%	1.56	(0.77; 3.25)	0.222	1.35	(1.10; 1.68)	0.004	1.42	(0.73; 2.84)	0.311
**Supervision (n=152)**	63%	2.70	(1.25; 5.97)	0.012	1.46	(1.15; 1.91)	0.003	2.09	(0.99; 4.53)	0.056

Table 3: impact of demographics on satisfaction: multivariable logistic models.
